# Preoperative Pulmonary Rehabilitation and Perioperative Outcomes in High-Risk COPD Patients Undergoing Lung Cancer Surgery: A Retrospective Cohort Study

**DOI:** 10.3390/diagnostics16071072

**Published:** 2026-04-02

**Authors:** Kubilay İnan, Onur Küçük, Merve Şengül İnan, Özgür Ömer Yıldız, Semih Aydemir

**Affiliations:** 1 Department of Thoracic Surgery, School of Medicine, Ankara Yıldırım Beyazıt University, 06370 Ankara, Türkiye; 2 Department of Anesthesiology and Reanimation, School of Medicine, Dokuz Eylül University, 35330 İzmir, Türkiye; 3 Department of Thoracic Surgery, Health Sciences University, Ankara Bilkent City Hospital, 06800 Ankara, Türkiye; 4 Department of Thoracic Surgery, Ankara Yıldırım Beyazıt University, Yenimahalle Training and Research Hospital, 06370 Ankara, Türkiye; 5 Department of Anesthesiology and Reanimation, Ankara Yıldırım Beyazıt University, Yenimahalle Training and Research Hospital, 06370 Ankara, Türkiye

**Keywords:** chronic obstructive pulmonary disease, lung cancer, pulmonary rehabilitation, perioperative outcomes, forced expiratory volume in one second, hospital length of stay

## Abstract

**Background/Objectives**: Chronic obstructive pulmonary disease (COPD) coexists with lung cancer in 40–70% of cases and increases perioperative risk, particularly in patients with severely impaired pulmonary function. Preoperative pulmonary rehabilitation (PR) has been proposed as a perioperative optimization strategy; however, its effect on hospital length of stay (LOS) in patients with advanced COPD remains unclear. This study aimed to compare postoperative complications, intensive care unit (ICU) utilization, and hospital LOS between patients with lower and higher baseline forced expiratory volume in one second (FEV1), and to evaluate the role of preoperative PR as a risk-adaptive perioperative strategy in high-risk COPD patients undergoing lung cancer surgery. **Methods**: This retrospective cohort study comprises patients with spirometry-confirmed COPD and non-small cell lung cancer (NSCLC) who underwent elective lung resection at a tertiary care center between March 2019 and June 2020. Disease severity was classified using the Global Initiative for Chronic Obstructive Lung Disease (GOLD) framework: GOLD 1–2 (FEV1 ≥ 50% predicted) and GOLD 3–4 (FEV1 < 50% predicted). Patients in the GOLD 3–4 group received a uniform 15-day hospital-based preoperative PR program prior to surgery. Primary outcomes were ICU stay, postoperative complications, and hospital LOS. Factors independently associated with prolonged hospital stay were examined using an exploratory multivariable linear regression model. **Results**: Among 63 patients (95.2% male; median age 64 years), those with GOLD 3–4 COPD had significantly lower baseline FEV1 values and longer COPD duration compared with the GOLD 1–2 group. Despite a higher perioperative risk profile, postoperative complication rates (28.6% overall; *p* = 0.237) and ICU utilization were comparable between groups. Median postoperative hospital LOS was significantly longer in patients with GOLD 3–4 COPD (15 [IQR 6] vs. 11 [IQR 4] days; *p* < 0.001). In the exploratory regression analysis, lower predicted FEV1 percent (*p* = 0.003) and older age were independently associated with prolonged hospital stay, whereas PR was not an independent determinant of LOS. **Conclusions**: In patients with lung cancer and severe COPD (GOLD 3–4) who received preoperative PR, postoperative complication rates and ICU utilization were comparable to those observed in patients with less severe disease. Prolonged hospital stay in the high-risk group was independently associated with lower FEV1 and older age, reflecting underlying disease severity. Prospective controlled studies stratified by COPD severity are needed to establish the independent contribution of preoperative PR in this population.

## 1. Introduction

Lung cancer is the leading cause of cancer-related death worldwide, accounting for approximately 2.2 million new cases and 1.8 million deaths annually [1]. Chronic obstructive pulmonary disease (COPD) frequently coexists with lung cancer, driven by shared etiological factors including tobacco exposure and chronic airway inflammation [2,3]. Epidemiological data indicate that COPD is present in 40–70% of patients diagnosed with lung cancer, independent of age, sex, and cumulative smoking history [3]. Beyond a coincidental association, COPD has been identified as an independent adverse prognostic factor in patients with resectable lung cancer, with hazard ratios for overall survival ranging from 1.4 to 2.4 after adjustment for age and smoking exposure [2,4]. Notably, even among never-smokers with COPD, in whom emphysema and lung cancer are less prevalent than in smoking-related disease, the oncological burden remains clinically relevant; non-exacerbating never-smokers with COPD have been reported to be more likely to die from malignancy than from respiratory causes [5]. These observations establish the coexistence of COPD and lung cancer as a distinct clinical challenge requiring specific perioperative strategies.

Surgical resection remains the primary curative treatment for early-stage non-small cell lung cancer (NSCLC). Reduced forced expiratory volume in one second (FEV1) in patients with coexisting COPD is associated with increased postoperative pulmonary complications, including atelectasis, pneumonia, and respiratory failure [6,7,8]. A recent meta-analysis of 93,805 patients identified COPD as the most consistent independent risk factor for postoperative pulmonary complications following lung cancer resection [9]. Current clinical practice guidelines, including the American College of Chest Physicians (ACCP) evidence-based recommendations, emphasize that reduced FEV1 should be interpreted as a marker of increased surgical risk rather than an absolute contraindication to resection, provided that appropriate perioperative optimization is implemented [7]. An international expert consensus on the management of lung cancer complicated by COPD reinforced this position, recommending preoperative pulmonary rehabilitation and bronchodilator optimization for patients with GOLD 3–4 disease prior to surgical reassessment [10]. Subotic et al. demonstrated that patients with COPD who underwent lobectomy experienced paradoxically less FEV1 loss than patients with normal lung function, a finding attributed to a lobar volume reduction effect [8]. These data support the concept that carefully selected patients with advanced COPD can undergo lung resection when adequate perioperative preparation is provided.

Pulmonary rehabilitation (PR) is a well-established, evidence-based intervention in COPD management. The 2013 American Thoracic Society/European Respiratory Society (ATS/ERS) statement defines PR as a comprehensive, patient-centered intervention incorporating exercise training, respiratory muscle training, and patient education [11]. Cochrane-level evidence demonstrates that PR improves exercise capacity (mean increase of 44 m in six-minute walk distance), reduces dyspnea, and enhances health-related quality of life in patients with COPD across all severity stages [12,13,14]. Given these established benefits in improving functional reserve, PR has been increasingly proposed as a preoperative optimization strategy for patients with lung cancer and coexisting COPD.

Several randomized trials and meta-analyses have demonstrated that preoperative PR reduces postoperative pulmonary complications (risk reduction of 58–68%) and may shorten hospital stay by 2–5 days in general lung cancer surgery populations [15,16,17,18,19,20,21]. However, the effect of PR on postoperative hospital length of stay (LOS) has been inconsistent. Three recent meta-analyses reported no statistically significant reduction in LOS following preoperative PR, with high heterogeneity across studies [22,23,24]. This discrepancy may reflect differences in baseline patient risk profiles, COPD severity, and rehabilitation protocols. Critically, no study to date has specifically examined the relationship between COPD severity stratified by GOLD classification and perioperative outcomes following preoperative PR in patients undergoing lung cancer surgery.

Given the absence of evidence examining perioperative outcomes stratified by COPD severity in patients receiving preoperative PR, a direct comparison of postoperative outcomes between GOLD severity groups is warranted. The aim of this study was to evaluate perioperative outcomes, including postoperative complications, ICU utilization, and hospital LOS, in patients with lung cancer and COPD stratified by disease severity following preoperative PR. We hypothesized that patients with severe COPD (GOLD 3–4) could undergo surgical resection following preoperative PR without an excessive increase in postoperative complications, despite a longer hospital stay reflecting underlying disease severity.

## 2. Materials and Methods

### 2.1. Study Design and Ethical Approval

This retrospective cohort study was conducted at Ankara Bilkent City Hospital, a tertiary referral center, following approval by the institutional clinical research ethics committee (approval date: 25 June 2020; reference number: E1-20-817). Study data were retrieved from the hospital’s electronic medical record database. All procedures complied with the principles of the Declaration of Helsinki (2024 revision) and internationally accepted ethical standards for human research. The requirement for written informed consent was waived by the ethics committee owing to the retrospective nature of the study. This manuscript was prepared in accordance with the Strengthening the Reporting of Observational Studies in Epidemiology (STROBE) guidelines for cohort studies.

### 2.2. Patient Population

The study screened all adult patients who had elective lung resection for lung cancer at our institution from March 2019 through June 2020. To be eligible, patients had to meet all of the following criteria: age 18 years or older; histopathologically verified NSCLC; clinical or pathological disease stage I through IIIA; and a spirometric diagnosis of COPD defined by a post-bronchodilator FEV1/FVC ratio below 0.70.

**Figure 1:**
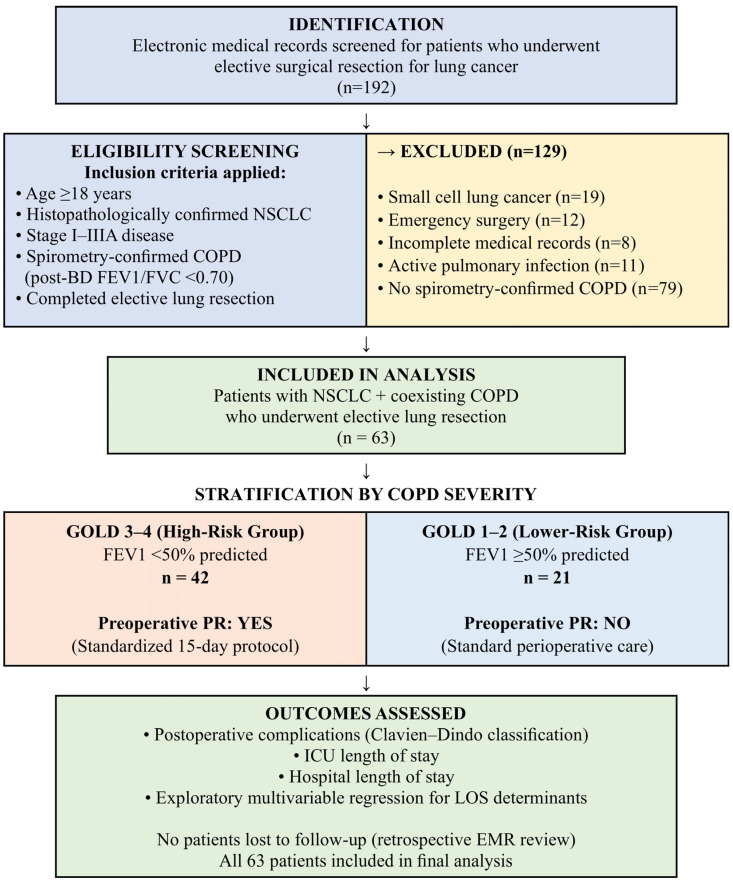
STROBE flow diagram of patient (BD, bronchodilator; COPD, chronic obstructive pulmonary disease; EMR, electronic medical record; FEV1, forced expiratory volume in one second; FVC, forced vital capacity; GOLD, Global Initiative for Chronic Obstructive Lung Disease; ICU, intensive care unit; LOS, length of stay; NSCLC, non-small cell lung cancer; PR, pulmonary rehabilitation.).

Patients were not included if surgery was performed on an emergency basis, if medical records were incomplete, if an active pulmonary infection was present in the preoperative period, or if the histological diagnosis was small cell lung cancer. The last exclusion was applied because small cell lung cancer follows a different biological course and is managed with distinct treatment protocols in which elective resection and preoperative PR have no established role. A total of 63 patients met all criteria and formed the final study cohort (Figure 1).

### 2.3. Group Stratification

Patients were stratified according to baseline pulmonary function using preoperative FEV1 percent predicted values, in line with GOLD severity classification. Patients with GOLD stage 1–2 COPD (FEV1 ≥ 50% predicted) were categorized as the lower-risk group, whereas patients with GOLD stage 3–4 COPD (FEV1 < 50% predicted) were classified as the high-risk group. All patients in the high-risk group received a standardized preoperative PR program prior to surgery, applied selectively based on routine clinical indication in accordance with ACCP guideline recommendations for patients with predicted postoperative FEV1 or DLCO below 60% [7]. Patients in the lower-risk group did not receive structured preoperative rehabilitation as part of standard perioperative care.

### 2.4. Pulmonary Rehabilitation Protocol

Preoperative PR was implemented as a standardized, hospital-based program with a fixed duration of 15 consecutive days prior to surgery. The program was administered five days per week and consisted of supervised daily sessions lasting 30–45 min. Rehabilitation components included aerobic exercise training (cycling and treadmill walking) tailored to each patient’s functional capacity, inspiratory muscle training using threshold loading devices, and structured patient education focusing on breathing techniques, airway clearance strategies, and perioperative respiratory care. This protocol duration and composition are consistent with the ATS/ERS statement on pulmonary rehabilitation [11] and the international expert consensus recommending approximately two weeks of preoperative PR for patients with lung cancer and COPD [10].

### 2.5. Data Collection

Demographic and clinical data collected from medical records included age, sex, body mass index (BMI), and smoking history expressed as pack-years. Pulmonary and disease-related variables included duration of COPD diagnosis, preoperative FEV1 values expressed both in liters and as percent predicted, tumor location and size, and maximum standardized uptake value (SUVmax). Surgical variables included the surgical approach, categorized as video-assisted thoracoscopic surgery (VATS) or open thoracotomy and type of lung resection performed.

### 2.6. Postoperative Complications

Postoperative complications were categorized using the Clavien–Dindo classification, a severity-based system that stratifies complications according to the level of therapeutic intervention required. Minor complications were defined as those managed without invasive procedures or with standard pharmacological treatment (grades I–II), whereas major complications required surgical, endoscopic, or radiological intervention, intensive care management, or resulted in death (grades III–V). For each patient, the most severe complication documented during the index hospitalization was used for analysis.

Given the study population, postoperative pulmonary complications were also specifically captured and included pneumonia, clinically significant atelectasis requiring bronchoscopic or non-bronchoscopic intervention, respiratory failure requiring non-invasive ventilation or reintubation and mechanical ventilation, bronchospasm requiring escalation of bronchodilator therapy, pleural effusion requiring drainage, and acute respiratory distress syndrome, when documented in the medical record. These events were captured as a composite pulmonary complication outcome rather than individually.

### 2.7. Outcome Measures

The predefined postoperative outcomes were postoperative ICU stay (days), postoperative hospital LOS (days), and postoperative complications. All outcomes were assessed during the index hospitalization by reviewing daily progress notes, nursing records, laboratory and radiology reports, and discharge summaries.

### 2.8. Statistical Analysis

Analyses were carried out with Jamovi (version 2.3.28; Sydney, Australia). The Shapiro–Wilk test was used to evaluate distributional normality. Normally distributed continuous variables are expressed as mean with standard deviation (SD); skewed variables are reported as median with interquartile range (IQR). Categorical variables are presented as counts (*n*) and percentages (%).

Between-group comparisons of continuous variables used Student’s *t*-test or the Mann–Whitney U test, depending on data distribution. Categorical variables were assessed with the chi-square test or Fisher’s exact test. An exploratory multivariable linear regression model was built to examine predictors of postoperative hospital LOS. Covariates entered the model on clinical grounds and included age, body mass index, FEV1 percent predicted, tumor size, operation time, and surgical approach. COPD severity group and PR exposure were not entered as separate covariates owing to their collinearity with FEV1 percent predicted. COPD duration was similarly excluded for the same reason. Given the sample size of 63 patients and six covariates, the model should be interpreted as exploratory. Regression coefficients (β) reflect the estimated change in LOS in days per unit increase in each predictor. Although postoperative LOS showed a right-skewed distribution, linear regression was chosen for its clinical interpretability in this exploratory context. Residual plots and Q-Q plots showed an acceptable fit, with minor deviations in the upper tail linked to a small number of patients with unusually prolonged stays. A two-sided *p*-value below 0.05 was considered statistically significant. No a priori sample size calculation was conducted, as the cohort comprised all consecutive eligible patients treated within the defined study period.

## 3. Results

A total of 63 patients met the inclusion criteria and were included in the analysis. The majority were male (*n* = 60, 95.2%), and the median age was 64 years (IQR 9). The median BMI was 29.4 kg/m^2^ (IQR 6.06), and the median smoking exposure was 48 pack-years (IQR 9.5). The median duration of COPD was 5 years (IQR 3). The median preoperative FEV1 was 1.45 L (IQR 0.49), with a median FEV1 percent predicted of 46.9% (IQR 11.8).

Patients were stratified according to COPD severity as GOLD 1–2 (*n* = 21; FEV1 ≥ 50% predicted) and GOLD 3–4 (*n* = 42; FEV1 < 50% predicted). Age, sex, BMI, and smoking history were comparable between the two groups. As expected, patients with GOLD 3–4 COPD had a significantly longer duration of COPD and lower preoperative FEV1 and FEV1 percent predicted values compared with patients with GOLD 1–2 COPD. ASA physical status differed significantly between groups; ASA class II was present exclusively in the GOLD 1–2 group, while ASA class IV was observed only in the GOLD 3–4 group (*p* < 0.001). Baseline demographic and clinical characteristics are summarized in Table 1.

There was no significant difference between groups with respect to surgical approach or resection extent; thoracotomy was the most commonly performed approach in both groups (71.4%), and lobectomy was the predominant resection type (84.1%), with wedge resections accounting for the remainder (*p* = 0.983). Median operation time was comparable between groups. Postoperative ICU stay was short in both groups, with a median duration of 2 days (IQR 1). Although ICU stay was statistically longer in patients with GOLD 3–4 COPD, the absolute difference was limited and was not accompanied by higher postoperative complication rates.

Median postoperative hospital LOS was significantly longer in patients with GOLD 3–4 COPD compared with those with GOLD 1–2 COPD (15 [IQR 6] vs. 11 [IQR 4] days; *p* < 0.001). Postoperative complications occurred in 28.6% of patients overall, with no significant difference between groups (*p* = 0.237). Of the 18 patients with complications, 14 (77.8%) had minor complications (Clavien-Dindo grade I–II) and 4 (22.2%) had major complications (grade ≥IIIa), with no significant difference between GOLD severity groups (*p* = 0.586). Surgical and postoperative outcomes are presented in Table 2.

In an exploratory adjusted linear regression model, lower FEV1 percent predicted was independently associated with prolonged postoperative hospital LOS; a 10% decrease in FEV1 corresponded to an approximately 1.9-day increase in hospital stay (*p* = 0.003). Older age was also significantly associated with longer hospital stay, whereas other perioperative variables showed no significant association (Table 3).

All patients in the GOLD 3–4 group had undergone preoperative PR. Despite significantly impaired baseline pulmonary function and longer COPD duration, patients with GOLD 3–4 COPD demonstrated postoperative complication and ICU utilization rates comparable to those observed in the lower-risk GOLD 1–2 group. There was no 30-day mortality in either group.

## 4. Discussion

In this retrospective cohort study of 63 patients with NSCLC and coexisting COPD, patients with high-risk disease, characterized by severely reduced baseline pulmonary function (GOLD 3–4), underwent surgical resection following preoperative PR without an excessive increase in postoperative complications or ICU utilization. Despite markedly lower FEV1 values and a higher perioperative risk profile, postoperative complication rates and ICU requirements remained comparable between GOLD severity groups, suggesting that preoperative optimization strategies may contribute to safe perioperative management in this vulnerable population.

The frequent coexistence of lung cancer and COPD reflects shared pathophysiological mechanisms beyond their common association with tobacco exposure. Chronic inflammation, oxidative stress, NF-κB pathway activation, and epigenetic alterations contribute to both airway remodeling and carcinogenesis, creating a two- to six-fold increase in lung cancer risk among patients with COPD [3,25]. COPD has been consistently identified as an independent adverse prognostic factor in resectable lung cancer. Wang et al. reported significantly reduced five-year overall survival in a cohort of 2222 lung cancer patients with coexisting COPD compared with those without COPD (65.2% vs. 73.3%), with the emphysema-predominant phenotype carrying the highest risk (HR 2.53 for squamous cell carcinoma) [2]. Zhai et al. confirmed these findings in 902 surgically resected early-stage NSCLC patients, demonstrating that COPD was an independent predictor of both reduced overall survival (adjusted HR 1.41; *p* = 0.002) and reduced progression-free survival (adjusted HR 1.67; *p* = 0.003) [4]. A meta-analysis of 93,805 patients identified COPD as the most consistent risk factor for postoperative pulmonary complications following lung cancer surgery, with a pooled relative risk of 1.72 [9]. Our results, consistent with their greater disease burden, indicated that patients in the GOLD 3–4 group had significantly higher ASA physical status scores compared with the GOLD 1–2 group (*p* <0.001); despite preoperative optimization, this group also required longer postoperative recovery, reflecting the cumulative impact of severe airflow limitation and its systemic consequences on perioperative physiology. The clinical implication is that perioperative risk stratification in these patients should incorporate COPD severity as a distinct prognostic variable rather than treating COPD as a binary covariate.

Preoperative PR has been shown to reduce postoperative pulmonary complications across multiple meta-analyses. Gravier et al. reported that preoperative exercise training reduced overall complications (RR 0.58; 95% CI: 0.45–0.75) and clinically significant complications (Clavien-Dindo ≥ 2; RR 0.42; 95% CI: 0.25–0.69) in 617 NSCLC patients across 10 studies [18]. The Cochrane review by Cavalheri and Granger, although limited to five RCTs with 167 patients, demonstrated a 67% reduction in postoperative pulmonary complications (RR 0.33; 95% CI: 0.17–0.61; number needed to treat of 4) [21]. Chen et al. confirmed a similar magnitude of pulmonary complication reduction (OR 0.33; 95% CI: 0.22–0.50) in a meta-analysis of 1338 patients [19]. Li C et al., in the most recent and largest meta-analysis of 16 RCTs with 1022 patients, reported a 67% reduction in overall postoperative complications (OR 0.33; 95% CI: 0.24–0.46) and a 65% reduction in severe complications (OR 0.35; 95% CI: 0.21–0.56) [16]. Of note, a meta-analysis restricted exclusively to patients with concurrent lung cancer and COPD reported a comparable reduction in postoperative pulmonary complications (OR 0.21; 95% CI: 0.12–0.37) and a 2.13-day decrease in hospital LOS in the RCT subgroup (I^2^ = 0%), providing COPD-specific meta-analytic support for these findings [26]. In our cohort, postoperative complication rates were comparable between the GOLD 3–4 and GOLD 1–2 groups (*p* = 0.237), despite markedly different baseline pulmonary function. This observation is consistent with preoperative PR having contributed to perioperative risk attenuation in the high-risk group; however, because all GOLD 3–4 patients received PR and no GOLD 1–2 patients did, the effect of PR cannot be separated from underlying disease severity or from the overall perioperative care provided to this group. Careful patient selection and multidisciplinary perioperative management may have contributed equally to the observed outcomes. Saito et al. reported analogous findings in a retrospective cohort of 116 NSCLC patients with COPD, where preoperative PR (mean duration 18.7 ± 12.7 days) was independently associated with reduced postoperative pulmonary complications after propensity score matching [27]. However, that study analyzed COPD as a single binary variable without stratification by disease severity, and the wide variation in PR duration (range 6–31 days) limited the ability to assess the contribution of a standardized protocol. Our study extends these findings by stratifying outcomes according to GOLD classification and applying a fixed 15-day PR protocol, thereby allowing a more precise evaluation of the relationship between COPD severity and perioperative outcomes in patients receiving uniform preoperative optimization.

The effect of preoperative PR on postoperative hospital LOS has been inconsistent across the literature. Earlier meta-analyses suggested substantial reductions: Sebio Garcia et al. reported a mean decrease of 4.83 days (95% CI: −5.90 to −3.76) [15], Ni et al. reported 4.98 days (95% CI: −6.22 to −3.74) [20], and the Cochrane review estimated 4.24 days (95% CI: −5.43 to −3.06) [21]. However, more recent and larger meta-analyses have not confirmed this finding. Guo et al. found no significant LOS reduction in 11 RCTs with 1250 patients (MD −0.23 days; *p* = 0.58; I^2^ = 83%) [23], and Cruz Mosquera et al. reported a similar null result (MD −0.91 days; *p* = 0.06; I^2^ = 71%) [22]. Xu et al. identified that the number of supervised sessions, rather than program duration, was the primary determinant of LOS reduction (β = −0.17), and that postoperative rehabilitation did not affect LOS [24]. In our study, median postoperative LOS was 15 days in the GOLD 3–4 group compared with 11 days in the GOLD 1–2 group. The regression analysis demonstrated that this difference was driven by lower FEV1 and older age rather than by PR exposure, supporting the interpretation that prolonged LOS in severe COPD reflects disease severity rather than treatment failure. Duan et al. identified patients with predicted postoperative FEV1 below 60% as the primary source of heterogeneity in LOS analyses across VATS-based studies, yet excluded this subgroup from further evaluation [28]. Our study directly addresses this gap by focusing exclusively on the high-risk population that drives heterogeneity in the existing literature, and by demonstrating that prolonged LOS in these patients persists despite preoperative PR, in the context of an open thoracotomy-predominant cohort (71.4%), which inherently experiences longer recovery trajectories than VATS-based populations.

The 15-day preoperative PR protocol used in the present study is consistent with current evidence supporting short-duration, high-intensity programs. Guo et al. reported that programs with a duration of three weeks or shorter were more effective than longer protocols in their subgroup analysis [23]. Lai et al. demonstrated that a seven-day inpatient PR program significantly reduced both postoperative complications (9.8% vs. 28.0%; *p* = 0.019) and postoperative LOS (6.1 vs. 8.7 days; *p* = 0.001) in 101 NSCLC patients at risk for pulmonary complications [29]. Brat et al., in a multicenter RCT of 122 high-risk patients defined by elevated VE/VCO_2_ slope, reported that a 14-day multimodal prehabilitation program reduced postoperative complications (34% vs. 55%; OR 2.29; *p* = 0.029) and LOS (7 vs. 9 days; *p* = 0.038) [30]. The LOS difference between our GOLD 3–4 group (median 15 days) and the populations studied by Lai and Brat (6–9 days) likely reflects the substantially greater baseline pulmonary impairment in our cohort rather than protocol inadequacy. Cho et al. similarly found no significant LOS difference (median 4 vs. 3 days; *p* = 0.051) after multimodal prehabilitation in 294 propensity-matched lung cancer surgery patients, despite significant reductions in overall and major complications [31]. Licker et al., in an RCT of 151 patients receiving preoperative high-intensity interval training, also reported no LOS difference between groups, while identifying COPD as an independent predictor of pulmonary complications (*p* = 0.043) [32]. The duration and content of our protocol are also consistent with an expert consensus developed specifically for patients with lung cancer and coexisting COPD, which recommends approximately two weeks of preoperative PR at five sessions per week, incorporating aerobic training, inspiratory muscle training, and patient education [33]. Collectively, these findings suggest that preoperative PR effectively reduces postoperative morbidity but does not consistently shorten hospital stay, particularly in patients with severe baseline pulmonary impairment.

The relationship between baseline functional capacity and postoperative recovery duration provides an important framework for setting realistic expectations in this population. Weinstein et al. demonstrated a strong inverse relationship between preoperative exercise capacity and postoperative LOS in 191 thoracic cancer surgery patients, with those in the lowest functional group (≤4 MET) requiring nearly twice the hospital stay of the highest group (9.2 vs. 4.8 days; *p* < 0.003) [34]. Given that patients with GOLD 3–4 COPD typically fall within the lowest functional strata, prolonged LOS should be anticipated rather than interpreted as a marker of treatment failure. Li X et al., in the only meta-analysis to separately analyze a COPD subgroup, reported a 6.73-day LOS reduction in 99 COPD patients who received preoperative exercise compared with untreated controls (95% CI: −9.88 to −3.58) [17]; however, this comparison assessed the effect of PR versus no PR, whereas our study compared outcomes across COPD severity strata in patients who all received perioperative care appropriate to their risk level. This distinction is critical: the relevant clinical question for patients with GOLD 3–4 disease is not whether PR shortens LOS compared with no intervention, but whether PR enables safe surgical treatment with acceptable morbidity despite an inherently longer recovery period.

This study has several limitations that warrant consideration. First, the retrospective, single-center design introduces the potential for selection bias, as patients with more severe pulmonary impairment were systematically selected to receive preoperative PR; this non-random treatment allocation may overestimate the benefit of PR in the high-risk group or, conversely, mask additional benefits in less severe patients. Second, the relatively small sample size (*n* = 63) limits statistical power, particularly for the exploratory regression analysis, and increases the risk of both type I and type II errors. Third, the absence of a formal control group of GOLD 3–4 patients who did not receive PR precludes direct assessment of the independent effect of PR on outcomes, and confounding by indication cannot be excluded. Fourth, adherence to the rehabilitation program and post-rehabilitation functional parameters (such as change in six-minute walk distance or FEV1 after PR) were not systematically recorded, limiting the ability to assess dose–response relationships. Fifth, the exploratory linear regression model did not account for the right-skewed distribution of LOS; although residual diagnostics indicated approximate normality with minor upper-tail deviations, alternative modeling approaches such as log-transformation or generalized linear models may yield different estimates. Furthermore, the inclusion of six covariates in a sample of 63 patients raises concern about overfitting, and the model could not be adjusted for cardiovascular comorbidities or baseline functional status owing to the unavailability of these data. Sixth, comprehensive comorbidity data beyond ASA physical status classification were not available from institutional records; the potential confounding effect of unreported comorbidities such as coronary artery disease, heart failure, and renal disease on postoperative outcomes cannot be excluded. Finally, the predominance of open thoracotomy (71.4%) and the overwhelmingly male population (95.2%) may limit generalizability to centers with higher VATS utilization rates or more balanced sex distributions.

Future prospective, multicenter studies with larger sample sizes are needed to confirm these findings and to define the optimal patient selection criteria for preoperative PR stratified by COPD severity. Such studies should incorporate standardized post-rehabilitation functional assessments and longer-term follow-up to evaluate the sustained impact of PR on recovery trajectories. The integration of preoperative PR into enhanced recovery after surgery (ERAS) pathways for patients with severe COPD represents a promising avenue for investigation.

## 5. Conclusions

Patients with lung cancer and severe COPD (GOLD 3–4) who received preoperative PR demonstrated postoperative complication rates and ICU utilization comparable to patients with less severe disease, despite significantly impaired baseline pulmonary function. Prolonged postoperative hospital stay in the high-risk group was independently associated with lower FEV1 and older age rather than with PR exposure, indicating that LOS reflects underlying disease severity. These findings are consistent with preoperative PR serving as a component of a risk-adaptive perioperative care pathway in selected patients with advanced COPD undergoing lung cancer resection, though the absence of a PR-naive high-risk control group precludes causal inference. The need for COPD severity-stratified outcome reporting and prospective controlled evaluation of preoperative PR in this population remains.

## Figures and Tables

**Table 1:** Distribution of patients’ demographic and clinical characteristics by group.

		Population (*n* = 63)	GOLD 3–4 COPD (Group 1, *n* = 42)	GOLD 1–2 COPD (Group 2, *n* = 21)	*p*-Value
Age (year), median (IQR)		64 (9)	64.5 (8.75)	61 (11)	0.158
Sex, *n* (%)	Male	60 (95.2%)	40 (95.2%)	20 (95.2%)	1.000
	Female	3 (4.8%)	2 (4.8%)	1 (4.8%)	
BMI (kg/m^2^), median (IQR)		29.4 (6.06)	29.4 (5.43)	29.6 (9.26)	0.550
ASA, *n* (%)	II	6 (9.5%)	-	6 (28.6%)	**<0.001**
	III	54 (85.7%)	39 (92.9%)	15 (71.4%)	
	IV	3 (4.8%)	3 (7.1%)	-	
Smoking (package/year), median (IQR)		48 (9.5)	49.5 (8.75)	45 (10)	0.066
Duration of COPD (year), median (IQR)		5 (3)	7 (3)	3 (1)	**<0.001**
Preop. FEV1 (lt), median (IQR)		1.45 (0.49)	1.34 (0.29)	1.84 (0.18)	**<0.001**
Preop. FEV1 (%expected), median (IQR)		46.9 (11.8)	44 (6.66)	59.7 (9.93)	**<0.001**
Diameter of tumor (cm), median (IQR)		3 (1.1)	2.95 (1.08)	3 (1)	0.787
SUVmax, median (IQR)		7.93 (7.74)	8.15 (6.25)	7.93 (13)	0.240
Tumor location	Lower right	7 (11.1%)	4 (9.5%)	3 (14.3%)	0.955
	Middle right	2 (3.2%)	2 (4.8%)	-	
	Upper right	18 (28.6%)	12 (28.6%)	6 (28.6%)	
	Lower left	13 (20.6%)	9 (21.4%)	4 (19%)	
	Upper left	23 (36.5%)	15 (35.7%)	8 (38.1%)	

Continuous variables are expressed as median (IQR), while categorical variables are expressed as frequency (percentage). Continuous variables were compared using the Mann–Whitney U test, while categorical variables were compared using the Pearson chi-square test or Fisher’s exact test. Statistically significant *p*-values are in bold. ASA: American Society of Anesthesiologists, BMI: body mass index, COPD: chronic obstructive pulmonary disease, FEV1: forced expiratory volume in the first second, GOLD: global initiative for chronic obstructive lung disease, SUVmax: maximum standardized uptake value.

**Table 2:** Distribution of surgical and postoperative outcomes by group.

		Population (*n* = 63)	GOLD 3–4 COPD (Group 1, *n* = 42)	GOLD 1–2 COPD (Group 2, *n* = 21)	*p*-Value
Surgical approach, *n* (%)	Thoracotomy	45 (71.4%)	30 (71.4%)	15 (71.4%)	1.000
	VATS	18 (28.6%)	12 (28.6%)	6 (28.6%)	
Type of lung resection, *n* (%)	Lobectomy	53 (84.1%)	35 (83.3%)	18 (85.7%)	1.000
	Wedge resection	10 (15.9%)	7 (16.7%)	3 (14.3%)	
Operation time (min), median (IQR)		240 (92.5)	240 (88.8)	255 (110)	0.502
ICU stay (day), median (IQR)		2 (1)	2 (1)	2 (1)	**0.004**
Hospital LOS (day), median (IQR)		13 (5)	15 (6)	11 (4)	**<0.001**
Complication, *n* (%)	Yes	18 (28.6%)	14 (33.3%)	4 (19%)	0.237
	No	45 (71.4%)	28 (66.7%)	17 (81%)	
Clavien-Dindo classification	I–II, *n* (%)	14 (22.3%)	11 (26.2%)	3 (14.2%)	0.586
	≥IIIa, *n* (%)	4 (6.3%)	3 (7.1%)	1 (4.8%)	

Continuous variables are expressed as median (IQR), while categorical variables are expressed as frequency (percentage). Continuous variables were compared using the Mann–Whitney U test, while categorical variables were compared using the Pearson chi-square test or Fisher’s exact test. Statistically significant *p*-values are in bold. VATS: video-assisted thoracoscopic surgery, ICU: intensive care unit, LOS: length of stay.

**Table 3:** Exploratory adjusted linear regression model evaluating factors associated with postoperative length of stay.

Predictor	β Coefficient (Days)	Standard Error	95% CI		*p* Value
			Lower	Upper	
Age (years)	0.266	0.114	0.036	0.495	**0.024**
BMI (kg/m^2^)	0.051	0.140	−0.229	0.332	0.715
FEV1% predicted	−0.188	0.061	−0.3110	−0.065	**0.003**
Tumor size (cm)	−0.160	0.588	−1.340	1.018	0.786
Operation time (min)	0.001	0.014	−0.027	0.030	0.920
Surgical approach, Thoracotomy—VATS	2.436	1.872	−1.314	6.188	0.199

β coefficients represent absolute change in postoperative length of stay (days) per unit increase in continuous variables. The model was constructed to explore whether postoperative LOS differences were consistent with baseline respiratory risk rather than to estimate a causal effect of pulmonary rehabilitation. Statistically significant *p*-values are in bold. BMI: body mass index, FEV1: forced expiratory volume in the first second, VATS: Video-assisted thoracoscopic surgery.

## Data Availability

The datasets used and/or analyzed during the current study are available from the corresponding author on reasonable request.

## Abbreviations

The following abbreviations are used in this manuscript:

%	proportion
ACCP	American College of Chest Physicians
ATS	American Thoracic Society
BMI	body mass index
CI	confidence interval
COPD	chronic obstructive pulmonary disease
ERAS	enhanced recovery after surgery
ERS	European Respiratory Society
FEV1	forced expiratory volume in one second
FVC	forced vital capacity
GOLD	global initiative for chronic obstructive lung disease
HR	hazard ratio
ICU	intensive care unit
IQR	interquartile range
LOS	length of stay
MD	mean difference
*n*	number
NSCLC	non-small cell lung cancer
OR	odds ratio
PR	pulmonary rehabilitation
PPC	postoperative pulmonary complications
RR	relative risk
SD	standard deviation
STROBE	Strengthening the Reporting of Observational Studies in Epidemiology
SUVmax	maximum standardized uptake value
VATS	video-assisted thoracoscopic surgery
